# Neurological impact of HIV/AIDS and substance use alters brain function and structure

**DOI:** 10.3389/fmed.2024.1505440

**Published:** 2025-01-07

**Authors:** James Haorah, Samikkannu Malaroviyam, Hemavathi Iyappan, Thangavel Samikkannu

**Affiliations:** Department of Pharmaceutical Sciences, Irma Lerma Rangel College of Pharmacy, Texas A&M University, College Station, TX, United States

**Keywords:** HIV, drugs of abuse, microglia, CART, HANDs

## Abstract

Human immunodeficiency virus (HIV) infection is the cause of acquired immunodeficiency syndrome (AIDS). Combination antiretroviral therapy (cART) has successfully controlled AIDS, but HIV-associated neurocognitive disorders (HANDs) remain prevalent among people with HIV. HIV infection is often associated with substance use, which promotes HIV transmission and viral replication and exacerbates HANDs even in the era of cART. Thus, the comorbid effects of substance use exacerbate the neuropathogenesis of HANDs. Unraveling the mechanism(s) of this comorbid exacerbation at the molecular, cell-type, and brain region levels may provide a better understanding of HAND persistence. This review aims to highlight the comorbid effects of HIV and substance use in specific brain regions and cell types involved in the persistence of HANDs. This review includes an overview of post-translational modifications, alterations in microglia-specific biomarkers, and possible mechanistic pathways that may link epigenomic modifications to functional protein alterations in microglia. The impairment of the microglial proteins that are involved in neural circuit function appears to contribute to the breakdown of cellular communication and neurodegeneration in HANDs. The epigenetic modification of N-terminal acetylation is currently understudied, which is discussed in brief to demonstrate the important role of this epigenetic modification in infected microglia within specific brain regions. The discussion also explores whether combined antiretroviral therapy is effective in preventing HIV infection or substance-use-mediated post-translational modifications and protein alterations in the persistence of neuropathogenesis in HANDs.

## 1 Introduction

Recent advances in combined antiretroviral therapy (cART) have visibly reduced the levels of HIV infection, replication, and HIV/AIDS progression. Moreover, these advances have contributed to gaining a better understanding of central nervous system (CNS) viral invasion, persistence, and neuropathogenesis (1–4). It is evident that while cART can control viral replication, a cure for HIV/AIDS remains elusive due to the persistence of the HIV-1 latent reservoir in microglia and monocytes/macrophages, as well as the limited penetration of cART across the blood-brain barrier (BBB) and its confinement within the restricted skull cavity (5–8). However, it has been shown that there is a direct correlation between ART concentrations and HIV-1 viral load in the brain (9), which indicates that cART can penetrate the BBB and enter the brain. Penetration of cART into the brain has been shown to cause adverse effects, including neurological complications (10–12). An equally important concern regarding HIV infection is the multifaceted complications fueled by substance use, such as cocaine, amphetamine, alcohol, and opioids. Opioids alone consist of several drugs of abuse, such as morphine, heroin, buprenorphine, oxycodone, fentanyl, methadone, and nalorphine (12). The interactions of these drugs with HIV-1 and their mechanisms of action are different for each drug. This review focuses on the role of long-term substance use that could contribute to the development and progression of HANDs arising from immune cell suppression, neuroinflammation, and neurodegeneration. Although these risk factors contribute to the development of HANDs, there are currently limited discussions regarding the interactive role of substance use that may contribute to risk factors of HIV transmission, infection, and non-adherence to cART regimens.

One of the common factors in substance use that contributes to neuro-AIDS development is undoubtedly damage to the BBB, which promotes the infiltration of infected cells into the brain for the progression of HANDs (2). Substance use, such as alcohol, is indeed a risk factor for HIV infection (13, 14) that contributes to the reduction in CD4^+^ T cells in HIV patients (15, 16). The comorbid effects of substance use in HANDs include depressive symptoms (17), memory loss (18), increased neuropathy (19), and an elevated mortality rate among HIV/AIDS patients (20). Thus, the BBB disruption, oxidative stress, cytokine release, and neuroinflammation mediated by HIV infection and substance use are thought to be due to the persistence of HANDs (21–24). Moreover, the cure/prevention of this fatal disease is hampered by the persistence of the HIV reservoir. The stability of the latent HIV-1 reservoir is harbored in the central memory CD4^+^ T cell genotype, and the integrated HIV DNA is harbored in the transitional memory CD4^+^ T cell genotype (25, 26). Together with these CD4^+^ T cells, the persistence of HIV-1 latency and HIV reservoirs in microglia, macrophages, and inflammatory monocytes within the restricted skull cavity appear to play a crucial role in the recurrence of HIV-1 self-renewal, reinfection upon cART withdrawal (27–29), and perhaps the chronic prevalence of HANDs. In fact, the use of highly suppressive cART has significantly controlled HIV/AIDS into a manageable chronic disease (2), but the prevalence of HANDs remains at 40–50% among people with HIV (30–32). In addition to the neuropsychological diagnosis of HANDs, it is also crucial to delve deeper into the specific brain region harboring the cell types that are explicitly linked to HIV resurgence and neuropathogenesis of HANDs. Understanding this knowledge gap could pave the path for uncovering the underlying molecular and cellular mechanisms and facilitate successful viral purging, thus making the cell types linked to HIV resurgence more accessible targets for cART.

## 2 Brain region affected in HANDs

Despite advancements in antiretroviral therapy (ART), the prevalence of HIV-associated neurocognitive disorders (HAND) and grey matter atrophy remains concerningly high among people with HIV (PWH), even with effective viral suppression and restored CD4^+^ counts (30, 32). Grey matter atrophy predominantly affects regions such as the frontal and temporal cortices, hippocampus, and basal ganglia. These changes are driven by viral proteins (e.g., Tat, gp120), chronic neuroinflammation, oxidative stress, and neuronal dysregulation. Substance use disorders (SUDs) further exacerbate these effects, intensifying neurodegeneration in vulnerable brain regions.

Understanding the specific brain regions and cell types affected by HAND and SUDs is critical for developing targeted therapies. Neuroimaging studies using powerful magnetic resonance imaging (MRI), computed tomography (CT) scanning, and positron emission tomography (PET) scans have demonstrated a strong correlation between regional brain atrophy and cognitive impairments, offering valuable insights into the pathophysiology of HAND. This complex interplay underscores the urgent need for a deeper understanding of the mechanisms driving selective grey matter atrophy and its interactions with comorbid SUDs. Such knowledge is essential for designing effective therapeutic interventions to mitigate HAND and its associated cognitive deficits.

Neuroimaging techniques have claimed to have the advantage of confirming the diagnosis of HANDs because they can exclude other diseases that can mimic them (33), but it is reasonable to state that the integration of information gathered from neuropsychiatric testing, CSF analytical markers, and neuroimaging findings is expected to provide a better diagnosis. Neuroimaging techniques have observed brain atrophy in all brain regions in PWH, which include the frontal, subcortical, temporal, parietal, occipital, and cerebellum, as shown by the various studies cited below. However, there are also some conflicting results, particularly regarding the effects of HIV infection on the brain volume of PWH. The findings include a reduced brain volume (34, 35), an increased brain volume (36), and no change in brain amygdala volume (37–39). To this end, Israel et al. (40) identify two distinct patterns of grey matter atrophy in HIV: frontal and anterior cingulate cortex (ACC) atrophy, which is associated with disease progression, and caudate/striatum atrophy, which is linked to neurocognitive impairments. These regions play distinct roles in the development of HIV-associated neurocognitive disorders (HANDs). Furthermore, this model generates several testable predictions and provides a framework for understanding HANDs within the broader context of behaviorally based models in people with HIV. A consistent finding is frontal or subcortical atrophy, observed in HIV-1 infection with/without substance use, such as cocaine, methamphetamine, alcohol, or opioid drugs in HANDs/SUDs (37, 40–46). Most neuroimaging studies have employed image- or coordinate-based MRI meta-analyses (47, 48). The former used full statistical maps and the latter used the reported coordinates of peak locations from the available original studies. The regions-of-interest-based colocalization likelihood estimation (CLE) approach was introduced to quantify atrophy at specific brain regions in PWH and people without HIV, which improves the limitations of these two voxel-based approaches (40). These studies showed that the two distinct atrophied brain regions affected in adults infected with HIV were the frontal subcortical and caudate/striatum, which were clearly differentiated from those without HIV-infected controls. The hallmark of frontal subcortical atrophy was attributed to the early development of HANDs, while caudate/striatum atrophy was attributed to the progression of neurocognitive impairment in HANDs. These findings were in line with those of other MRI-based findings that showed that HIV infection selectively damaged the cortex, even during the period in which patients received cART (49). Further, coordinate-based MRI meta-analysis showed that caudate/striatum atrophy in HANDs correlated with a decrease in cortical thickness and impairment of neurocognitive function (48–50). Although most brain regions are affected by HANDs/SUDs, it is reasonable to state that frontal subcortical and caudate/striatum may be the prominent brain regions selectively affected and atrophied by HANDs/SUDs. Supporting these neuroimaging observations, neuropathological examinations of postmortem brain tissues from individuals with HIV infection and substance use have confirmed significant brain damage in the frontal cortex, substantia nigra, and cerebellum. This damage is characterized by synaptic and dendritic density loss and pronounced neuroinflammation (51–53). Further studies have corroborated these findings, noting an increased number of infected macrophages and microglia in the perivascular space and the presence of multinucleated giant cells—hallmark features of HIV-associated neurocognitive disorders (HANDs) (54, 55).

## 3 Specific cell types in affected brain regions

It is pertinent to identify whether microglia, astrocytes, or infiltrated monocytes/macrophages actively participate in promoting the propagation and progression of atrophy in HANDs in these two distinct brain regions affected by HIV infection and substance use. Together with the role of astrocytes and oligodendrocytes, microglial cells are considered one of the key players in maintaining brain homeostasis and protecting the well-being of neuronal function in the context of HIV infection. Microglia are the brain’s resident innate immune cells that act as immune surveillance, phagocytose the unhealthy dying cells, and scavenge dead cell debris or toxic proteins for the healthy maintenance of the brain environment. As such, microglial cells are well fortified with immune defense mechanisms, well-orchestrated neurotransmitter molecules, receptors for cellular communication, and xenobiotic sensors for the elimination of toxic molecules in the brain (56). Microglia and infiltrating inflammatory monocytes/macrophages are likely key drivers of neuroinflammation and neurodegeneration in the context of HIV infection and substance use disorders. HIV infection disrupts microglial function, and these effects are exacerbated by substance use, resulting in the release of various inflammatory cytokines and subsequent neuroinflammation, ultimately contributing to neurodegeneration. It is now well established that without the direct involvement of oligodendrocytes (57), the HIV-infected microglia, astrocytes, and monocytes/macrophages serve as the viral reservoir, viral latency, and viral resurgence cells in the CNS (30, 31, 58, 59). These factors serve as a roadblock to the eradication of HIV/AIDS and effective prevention of HANDs; therefore, microglia and monocytes/macrophages have attracted considerable interest in the study of the persistence of HANDs in substance use disorders. The rationale is that HIV-infected innate immune cells have been shown to increase the production of inflammatory cytokines TNF-α, IL-1β, IL-4, L-6, IL-2, IFN-γ, and IL-8 (60–64) in addition to the release of neurotoxic HIV proteins (65–67). Similarly, the role of microglia in the brain emerged as novel targets in opioid use research because microglia play an important role in shaping neural circuitry (68). Thus, identification of the impaired neurotransmitter molecules in the infected microglia is likely to afford a better strategy to protect these neurotransmitters for improved microglia-neuron communication in HIV/SUDs.

On the question of whether substance use impacts HIV infection, neuropathogenesis, and latency reversal, there seem to be contradictory reports, mostly on the use of opioids. Opioids such as morphine, fentanyl, and methadone facilitate HIV infectivity by suppressing antiviral genes and altering immune responses, particularly in CD4^+^ T cells and monocyte-derived macrophages (MDMs). This includes the upregulation of pro-inflammatory cytokines like TNF-α and IL-6, which promote a microenvironment conducive to HIV replication (69–71). Methadone, commonly used in opioid replacement therapy, compromises T cell responses, weakening host resistance to HIV (72, 73). Additionally, opioids modulate CXCR4, a co-receptor for HIV-1 entry, enhancing viral susceptibility in CD4^+^ T cells (74). Chronic opioid use increases latent HIV reservoirs, with studies showing higher viral loads and latency reversal, particularly in heroin users who inject drugs (70, 75, 76). These clinical and epidemiological findings were also supported by *in vitro* studies that demonstrated that HIV reactivation was observed mostly in opioid users (77).

Buprenorphine, a partial opioid agonist, increases HIV infection in CD4^+^ T cells but does not reactivate latent HIV-1 in resting CD4^+^ T cells, likely due to its unique pharmacological profile (78). Opioids also exacerbate HIV-associated neuropathogenesis through neuroinflammation and glial activation. Astrocytes and microglia are key players in these processes, contributing to neuronal injury and amplifying neurodegeneration in HIV-infected individuals (70, 77). Despite substantial evidence supporting the detrimental effects of opioids on HIV, some studies suggest that their immunosuppressive properties might reduce inflammation-driven replication, highlighting the complexity of these interactions (79). If HIV-infected monocytes fail to migrate into the CNS, the virus may remain in peripheral circulation, increasing systemic viral load as these cells act as reservoirs and release new virions. These monocytes may migrate to other tissues, such as the liver, spleen, or lymph nodes, establishing reservoirs or causing local inflammation. Reduced migration to the CNS may limit reservoir establishment in the brain, potentially decreasing direct CNS inflammation but allowing systemic inflammation to affect the CNS indirectly. Recent studies indicate that opioid use does not directly affect HIV replication, latency, or reactivation but indirectly shapes the HIV reservoir, reducing the reactivation of HIV latency (80, 81).

On the other hand, methamphetamine is considered mostly stimulatory by activating CD4 T cells, which has been suggested to be a contributing factor to HIV replication and disease progression (82, 83). Notably, methamphetamine exposure contributed to enhanced migration of HIV-infected monocytes into the brain for the establishment of HIV reservoirs, persistence, and a source of inflammation (84). Moreover, poor adherence to cART regimens by methamphetamine users contributed to HIV disease progression (85). Furthermore, methamphetamine exposure has been shown to suppress T cell proliferation by altering the G-phase cell cycle, thereby impairing the innate immune system (86). Moreover, methamphetamine treatment significantly suppressed the levels of IL-2, IFN-γ, IL-10, and MCP-1 production in mice (87, 88).

In contrast, cannabinoid THC has contradictory effects on CD4 T cells and intermediate monocytes. The use of THC, which is considered anti-inflammatory, in HIV infection is associated with lower T cell activation, a potential protective mechanism against HIV replication (89, 90). However, clinical studies have shown a significant increase in intermediate monocyte populations and a notable decrease in TNF-α levels in these cell types in cannabis users compared to controls (91, 92). Conversely, a substantial increase in the levels of cytokines IL-6, IL-8, TNF-α, and IL-10 has been observed in cannabis smokers (93). Nevertheless, cannabis may alter immune responses in ways that are not always beneficial, particularly if it affects the immune system’s ability to control HIV replication or repair immune dysfunction. Cannabis has been shown to reduce T cell activation and TNF-α levels in monocytes, potentially providing a protective mechanism against HIV replication. However, clinical studies have also reported an increase in intermediate monocyte populations among cannabis users, highlighting the complex and potentially dual role of cannabinoids in modulating immune responses during HIV infection.

## 4 Identification of cell phenotypes

Neuroimaging techniques have proven to be powerful tools for analyzing region-specific brain atrophy and the detection of microglia phenotypes in affected regions of the brain in HIV/SUDs. Among these techniques, positron emission tomography (PET) and MRI are widely utilized for assessing microglial inflammation. PET scanning has emerged as a potential technique for investigating neuroinflammation resulting from microgliosis. PET enables the identification of microglial phenotypes and the assessment of alterations in functional proteins within the identified phenotypes at the cellular and molecular levels, respectively (94, 95). Precision is achieved via PET by using radiotracers that target the proinflammatory M1 phenotype and various biomarkers at the cellular and molecular levels, respectively.

Microglia-specific biomarkers used in PET imaging include colony-stimulating factor 1 receptor (CSF1R), mitochondrial 18 kDa translocator protein, an array of purinergic receptors (such as the P2X7 receptor, involved in immune signaling and NLRP3 activation), cannabinoid receptor type 2, cyclooxygenase-2 (COX-2), and reactive oxygen species (ROS). For example, the PET radiotracer [^11^C] CPPC targeting the CSF1R marker in microglia has demonstrated increased uptake in murine and non-primate models of neuroinflammation (96), murine models of Alzheimer’s disease (AD) (97), AD postmortem brain tissues (98), epilepsy seizure imaging (99), and PET imaging of microglial activation in patients with multiple sclerosis or cerebellar ataxia in HIV infection (100, 101). Similarly, the use of an ^11^C-CB184 PET scan revealed elevated uptake of ^11^C-CB184 in cerebellar microglia of a gp120 mouse model of HIV infection (102) and in humans with HIV (103). Specifically, CSF1R, TSPO, P2X7, and CB2 are particularly relevant for imaging microglial activation in neuroinflammatory conditions like HIV. COX-2 and ROS are broader indicators of neuroinflammation involving various cell types and activated microglia. Identifying the ability of PET imaging to identify neuronal cells, astrocytes, oligodendrocytes, and microglial phenotypes is a significant achievement, unraveling the cellular mechanisms associated with HANDs (46, 94, 95). A recent article emphasized the importance of neuroimaging analysis in people with HIV (PWH), suggesting that an improved, common neuroimaging approach may enhance our understanding of CNS complications in PWH (104), potentially refining the characterization of HAND persistence. Emerging advancements in PET neuroimaging techniques targeting activated microglia contribute to a better understanding of neurochemical-stimulated neurophysiological alterations in HANDs (46).

In addition, the advancement in capturing the presence of macrophage/microglia M1/M2 phenotypes, CD^8+^ T cell CD^4dim^ CD^8bright^ T cell phenotypes, and astrocyte A1/A2 phenotypes in the restricted skull cavity, perivascular, and meningeal space provides an important predictor of HIV infection/invasion in the brain. This comprehensive assessment may contribute to the understanding of neuroinflammation, neurodegeneration, and persistence of HANDs in PWH. In the context of HIV and substance use disorders, increased expression of M1 macrophage or microglia phenotypes, the astrocyte type A1 subset, and CD8^+^ T cells in these brain regions would suggest HIV/SUD-induced neuroinflammation in HANDs and other neurological diseases (105, 106). In contrast, elevated expression of M2 microglia or macrophage phenotypes (107, 108), the astrocyte type A2 subset (109, 110), and the CD8^+^ T cell CD^4dim^ CD^8bright^ subset (111–113) would indicate an anti-inflammatory response to HIV neuro-invasion.

While HIV primarily infects and activates proinflammatory cell phenotypes in microglia and monocytes/macrophages, the question of astrocyte infection has been debated. Earlier studies demonstrated HIV infection in astrocytes by observing the integration of HIV-1 DNA in the astrocyte genome (114, 115). Most recent studies have concluded that astrocytes are not directly infected by HIV-1 in the brain but are activated by the virus. HIV infection in the brain occurs primarily in microglia and migrated monocytes/macrophages (116). It was previously believed that HIV infects only differentiated macrophages, not monocytes (117–119); however, recent findings have demonstrated that HIV can infect monocytes, which also act as reservoirs in the brain (120, 121). In light of these findings, it is safe to assert that neuroimaging techniques, particularly those utilizing PET radioligand binding methods, play a crucial role in assessing glial and monocyte inflammatory phenotypes during neuroinflammation (29, 122, 123). Therefore, delving into the underlying molecular mechanisms involved in inflammatory phenotypes becomes essential, which may offer insights into the supportive infection of bystander neuroimmune cells and potentially contribute to the severity of HANDs.

## 5 Molecular events in HIV/SUDs

While noninvasive neuroimaging techniques offer insights into immune cell response and longitudinal tracking in specific brain regions, understanding the molecular mechanistic events that may occur within the infected cells or activated cells surpasses the capabilities of neuroimaging techniques. It is well established that infected microglia or activated astrocytes produce a cascade of inflammatory cytokines, chemokines, ROS, and toxic HIV proteins (60–67). Concurrently, a decrease in anti-inflammatory cytokines and interferons is observed in both HIV infection (124, 125) and substance use disorders, such with alcohol, opioids, cannabinoids, and other drugs of abuse (126–128). Consequently, the production of proinflammatory agents outweighs the retention of anti-inflammatory molecules in HIV-infected or substance-exposed microglia. Therefore, it is crucial to understand the balance of these functional molecules within these affected cells during the inflammatory process.

Microglia, the brain’s innate immune cells, are equipped with immune surveillance, phagocytic scavenging capabilities, and functional receptor proteins that are essential for various neurophysiological functions. Some of these molecules that are predominantly expressed in microglia include ATP-dependent ionotropic purinergic receptor (P2X7R) (129, 130), metabotropic purinergic receptor (P2Y12R) (131, 132), colony-stimulating factor 1 receptor (CSF1R) (96, 133), cyclooxygenase-1/2 (134, 135), triggering receptor expressed in myeloid cells (TREM1) (136–138), cannabinoid type 2 receptor (CB2R) (139, 140), and γ-aminobutyric acid type B (GABA-B) receptors (141, 142). These biomarker receptors are mostly expressed in microglia and regulate cell proliferation, survival, differentiation, activation, and neurotransmission, and they also serve as sensors for apoptotic cell death. It should be noted that the above-cited biomarkers are not just limited to microglia; they are also expressed in other brain cell types, including monocytes/macrophages.

Recent findings highlighted that activation of microglia GABA-B receptor and CSF1R by astrocytic GABA release plays an important role in the microglia-neuron communication pathway (143, 144). While these microglia receptors remain inactive under normal physiological conditions, their significant up-regulation occurs during HIV-associated neuroinflammation (137, 145, 146). The activation of microglia and its receptors undoubtedly plays a vital role in the progression of neuroinflammation in HANDs and substance use disorders, such as with the long-term use of cocaine, morphine, amphetamine, alcohol, and opioids (146–149). Radiotracers that target these microglia receptors as biomarkers for inflammatory and neurodegenerative diseases have garnered considerable interest in the longitudinal visualization of disease progression (96, 128, 132, 135, 138). Beyond targeting these functional receptors, it is imperative to discuss the involvement of epigenomic, genomic, and transcriptomic factors in HIV/SUD-activated microglia that may contribute to the development of neuroinflammatory diseases.

## 6 Impact of epigenetics in HANDs

Recent interest has surged regarding research into understanding the effects of microglial epigenetic and transcriptomic modifications on the pathophysiology and neuropathogenesis of substance use disorders (69). Notably, these epigenetic or transcriptomic landscape modifications in microglial phenotypes exert control over neurophysiological functions and offer potential molecular mechanisms in neurological diseases (150, 151). This discussion focuses on the epigenetic modifications occurring in brain-resident microglia, which appear to exhibit a distinct epigenome, transcriptome, and chromatin landscape that differs from engrafted parenchymal brain macrophages (150), *ex vivo* cell cultures (152, 153), or microglia during the neurodevelopmental stage (154–156). It is rather challenging to distinguish between microglia and migrated macrophages in the brain because of the shared lineage and similar functional receptors (157, 158). To this end, a recently identified microglia-specific biomarker, transmembrane protein 119 (TMEM119), appears to be a promising authentic microglia-specific biomarker for cell phenotyping, as demonstrated by an investigation of transcriptional identity and developmental heterogeneity in single-cell sequencing (159–163). Recently, the chromatin landscape for HIV-1 integration determinants in microglia was identified at the CTCF-enriched domain (164), and region-specific epigenetic and transcriptomic alterations induced by HIV infection and substance use disorders in microglia were observed (165). It has been reported that HIV infection and substance use can modulate DNA methylation, histone modification, and chromatin conformation in microglia, with the underlying mechanisms of epigenetic alterations in human disease (164–168).

Advancements in various epigenetic modifications have led to a new therapeutic approach targeting the inhibition of these alterations as a potential cure for HIV/AIDS (169). Strategies include the “shock and kill” approach, aiming to shock the provirus into expression and then eliminate the cells by the immune system or neutralize them with antibodies (170); stimulating latently infected cells and challenging them with epigenetic inhibitors of HDACs, DNMTs, or combinations (171–173); and the “lock-and-block” approach, which focuses on silencing provirus reactivation using inhibitors of Tat (174), mTOR inhibitors (175), or inhibitors of an epigenetic reader BRD4 (176).

Epigenetic interventions for an HIV cure primarily revolve around the inhibition of lysine methylation or acetylation, given their demonstrated requirement for maintaining HIV-1 latency (177, 178). The role of N-terminal acetylation, catalyzed by N-terminal acetyltransferase (NAT), has been underexplored in HIV infection and substance use disorders. However, recent findings highlighted the critical role in regulating chromatin function and control of various gene expressions that influence functional protein dysregulation (179, 180). N-terminal acetylation has been implicated in HIV-host interactions and mitochondrial epigenetic modification as a cause of HIV neuropathogenesis (181). Activation of N-α-acetyltransferase 60 (NAT60) has been shown to promote influenza A viral infection, which inhibited the interferon-α signaling pathway (182), while NAT-B-mediated N-terminal acetylation has been shown to inactivate influenza virus PA-X and viral polymerase activity (183). Given the critical regulatory roles and emerging impact of N-terminal acetylation in health and disease, there is undoubtedly considerable interest and prospects for research exploring the pivotal role of NAT and its inhibitors, in particular, unraveling the epigenetic regulation of neurocognitive functional alterations in HANDs/SUDs.

## 7 Impact of antiretroviral therapy in HANDs

The introduction of highly active combination antiretroviral therapy (cART) has significantly transformed HIV/AIDS from a fatal to manageable chronic disease. However, the impact of cART on the prevalence of HANDs remains a subject of ongoing debate. The Frascati subclassification of HANDs encompasses asymptomatic neurocognitive impairment (ANI), minor neurocognitive disorder (MND/HAND), and severe HIV-associated dementia (HAD) (184). In the pre-cART era, HAD was associated with high viral loads and CD4 counts less than 200 cells/μL, while HANDs developed in patients with normal CD4 counts (30). In the cART era, there has been a substantial reduction in HAD, but the prevalence of HANDs remains noteworthy among people with HIV (30–32). Both HANDs and HAD share the same root cause, that is, HIV infection followed by chronic inflammation and neurotoxicity. The distinguishing factor appears to be viremia and CD4 counts, which are effectively managed in the cART era. Mild neurocognitive dysfunction in HANDs persists, suggesting that while cART effectively inhibits HIV replication, it may not entirely counteract the effects of shed neurotoxic viral proteins, secreted inflammatory agents, or ongoing oxidative stress in HIV patients. This persistent dysfunction may also be attributed to the “legacy effect,” where neuropsychological impairments result from brain damage caused by HIV infection during the pre-cART era, adverse side effects of cART, or comorbid substance use disorders (185, 186). Despite its success, cART has limitations in addressing the multifaceted aspects of HANDs. Although it effectively suppresses viral replication, cART exerts beneficial and adverse effects on neurocognitive function in HANDs. However, during the pre-cART era, HIV infection often led to more severe immune system damage, and many people with HIV developed neurological impairments such as HANDs due to the lack of effective viral suppression (10); however, this did not occur without HIV in people with SUDs. We propose that chronic inflammation, driven by persistent HIV resurgence and compounded by the adverse impact of cART and substance use disorders, contributes to the persistence of mild neurocognitive dysfunction in HANDs. However, there is limited evidence of neurocognitive effects or brain atrophy from PrEP use, as most studies focus on its efficacy for HIV prevention. Neurological impacts have been observed in ART users, such as cognitive impairments and brain changes, but these findings are not well documented in PrEP users without HIV.

Concerns arise from the broad spectrum of adverse effects associated with cART, including renal toxicity, mitochondrial metabolic impairment, gastrointestinal symptoms, cardiovascular effects, hypersensitivity, skin reactions, insomnia, and neuropsychiatric symptoms (187–189). These side effects often lead to interruptions in cART adherence by people with HIV (190). It is well documented in the literature that long-term cART use alone is associated with reduced levels of brain-derived neurotrophic factor (BDNF), astrogliosis, and increased release of proinflammatory cytokines (IL-1β and TNF-α), suggesting potential contributions to HAND development even in the absence of HIV infection (191). The co-occurrence of substance abuse in HIV infection further complicates cART efficacy and exacerbates HAND progression (54). The co-occurrence of substance abuse with HIV infection further complicates cART efficacy and exacerbates HAND progression (192). Regarding the ability of cART to penetrate the blood-brain barrier, evidence suggests that while cART affects brain functions, supporting its potential penetration into the CNS, its pharmacokinetics vary. Some cART drugs are metabolized quickly within the CNS, whereas others, particularly those with high CNS penetration effectiveness (CPE) scores, may persist longer. Furthermore, cART metabolites or low concentrations of these drugs can remain in the CNS for weeks to months post treatment. However, their functional effects, such as residual toxicity or viral suppression, may differ. Adverse effects on BDNF levels, induction of astrogliosis, upregulation of proinflammatory cytokines, and resurgence of HIV in microglia upon cART interruption reinforce the complex interplay between cART, HIV, and HANDs. The persistence of HIV infection in CNS reservoir cells and chronic inflammation may significantly contribute to HAND development. To effectively manage HANDs in the context of substance use, it is crucial to target HIV reservoir cells in the CNS with improved cART to prevent HIV resurgence. HIV reservoirs in the CNS primarily include microglia and astrocytes, while neurons are indirectly affected by inflammation and viral proteins. Improved long-acting cART aims to enhance CNS penetration and reduce dosing frequency. However, it faces challenges with HIV latency and potential resurgence, increasing the risk of neuroinflammation and cognitive decline. Additionally, exploring adjuvant therapeutic approaches to mitigate chronic neuroinflammation and neurotoxicity resulting from cART adverse effects, toxic viral proteins, secreted proinflammatory agents, and continuous oxidative stress remains a critical avenue for future research.

Current understandings of CNS HIV reservoirs focus predominantly on microglia, but this perspective is limited. Astrocytes also play a critical role as reservoirs, contributing to viral persistence and CNS dysfunction. While neurons are not directly infected, they experience indirect effects such as neurotoxicity, inflammation, and oxidative stress. Expanding research beyond microglia is essential to fully understand and address the multifaceted impact of HIV in the CNS.

## 8 Concluding remarks and future perspectives

**Figure 1:**
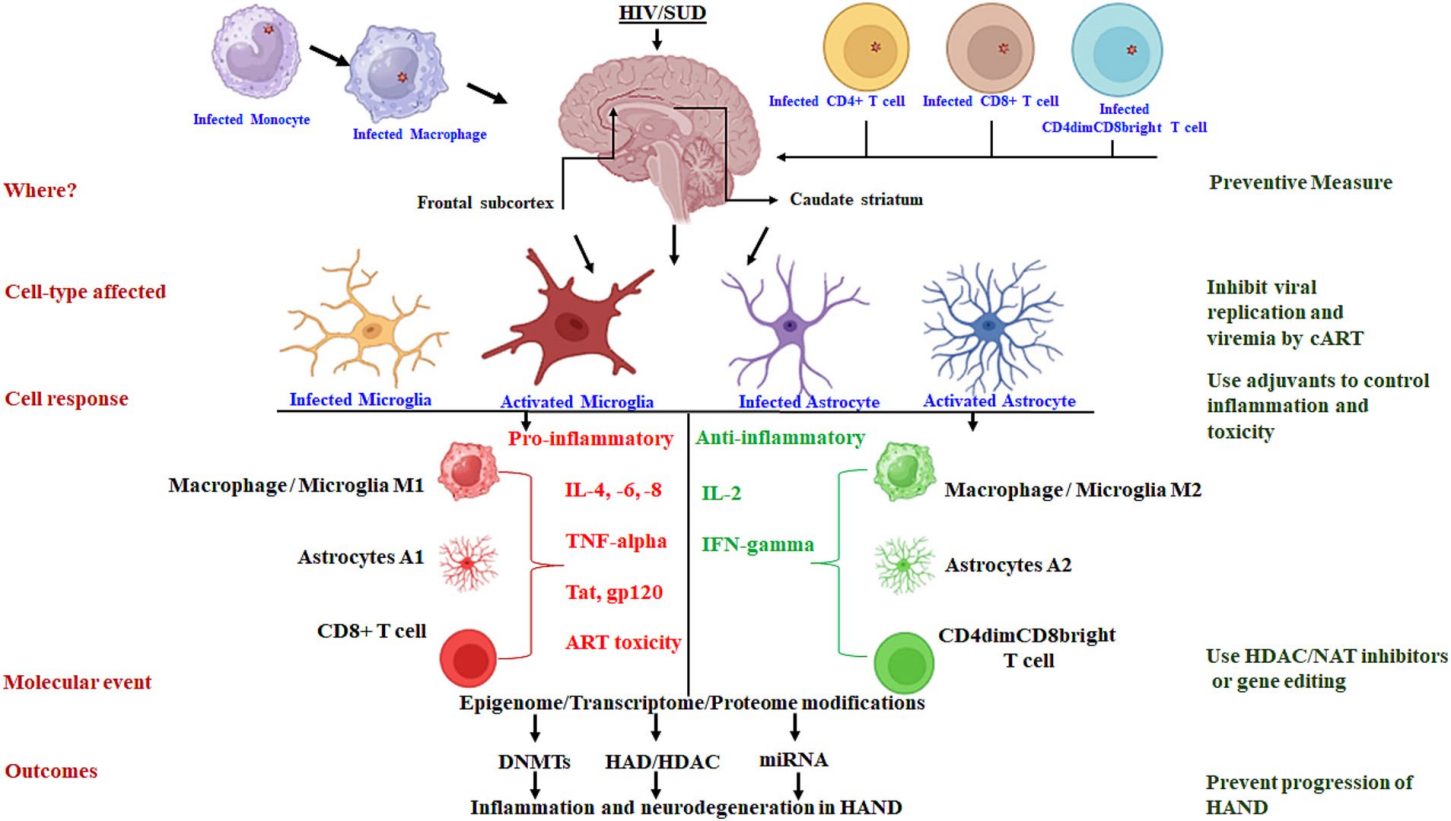
Schematic presentation of the molecular mechanisms driving chronic inflammation in brain-region-specific and cell-type-specific contexts: targets for preventive approaches.

In the complex landscape of HANDs, the coexistence of SUDs adds layers of complexity to the neuropathogenesis, potentially through oxidative stress and prolonged inflammatory processes driven by microglia phenotypes and migration of HIV-infected monocytes in specific brain regions. In the era of combination antiretroviral therapy (cART), it is crucial to delve into the brain-region-specific and cell-type-specific molecular mechanisms triggering chronic inflammation to develop preventive approaches to each of the mechanistic events associated with the persistence of HANDs, as described in Figure 1. Neuroimaging techniques have provided evidence that frontal subcortical and caudate striatum atrophies have been linked to the initial development of HIV disease and subsequent neurocognitive impairment in HANDs. Neuroimaging methods are believed to have the advantage of differentiating HANDs from other diseases that might mimic their symptoms, even in the presence of cART. Therefore, improvements to combined imaging tools offer hope for the accurate identification of specific brain regions affected in HANDs/SUDs and the specific types of cells that are involved in the progression of chronic inflammation within the identified brain region(s).

Microglia, as brain-resident cells, emerge as key players in CNS HIV infection, serving as viral reservoirs and potential sources of inflammation for the propagation of chronic neuroinflammation. Recent findings showed that substance use exacerbates immune activation, neuroinflammation, and mitochondrial dysfunction, and increases HIV replication, which are intimately linked to the pathology of HANDs. Thus, the identification of inflammatory microglia phenotypes in specific brain areas through PET imaging and targeting of microglial-specific biomarkers hold promise in understanding HAND prevalence in people with HIV. As such, microglia are significant contributors to HAND persistence in the context of substance use. Thus, exploring the molecular mechanisms and the contributing factors of substance use in HAND prevalence through microglial epigenetic modifications studies may be an important step to unravel the inflammatory phenotypes for inducing neurophysiological defects. The distinct epigenome, transcriptome, and chromatin landscape of microglia is of prime research interest. In particular, the epigenetic regulation and reprogramming of the “innate immunity” of brain-resident cell microglia and monocyte-differentiated macrophage brain homing cells can address the knowledge gap in the development and progression of HANDs. In this regard, the focus of research will be to evaluate whether interruption and re-adherence of cART in chronic HIV infection will impact trained/tolerized microglia/macrophages more during epigenetic modifications and metabolic switch, which are the two pillars of reprogramming innate immunity. Interest in epigenetic interventions for a HIV cure, such as inhibiting modifications, “shock and kill,” or reprogramming of innate immunity strategies, has increased with recent findings that highlight the crucial role of mitochondrial N-terminal acetylation in HIV-host interactions.

On the cART front, new antiviral drugs and immune activation therapies specifically targeting the latently infected cells (microglia/macrophages/monocytes that serve as HIV reservoirs) could bridge the current knowledge gap. Such drug and therapy advancements could include the formulation of improved antiretroviral drugs that can easily penetrate the blood–brain barrier, tissue-specific cART delivery, and purging of latently infected cells from anatomical HIV reservoirs. Despite successful viral load suppression by cART, the persistence of latent HIV reservoirs in the CNS impedes the possibility of HIV eradication. Chronic neuroinflammation and neurotoxicity from both latently infected microglia HIV reservoirs and cART adverse effects are suspected to be the factors contributing to HAND persistence. In conclusion, until the development of a new, improved, and less toxic antiviral drug, there is an urgent need for neuroprotective and anti-inflammatory adjuvant therapies along with cART regimens to control HAND progression in chronic HIV disease.
